# Mind wandering in sensory cortices

**DOI:** 10.1016/j.ynirp.2021.100073

**Published:** 2021-12-17

**Authors:** Shao-Min Hung, Po-Jang Hsieh

**Affiliations:** a Biology and Biological Engineering, California Institute of Technology, Pasadena, CA, USA; b Neurosciences, Huntington Medical Research Institutes, Pasadena, CA, USA; c Neuroscience and Behavioural Disorders Programme, Duke-NUS Medical School, Singapore; d Department of Psychology, National Taiwan University, Taipei, Taiwan

**Keywords:** Mind-wandering, Phenomenology, Sensory cortices, Support vector machine

## Abstract

The recent task-free approach in Cognitive Neuroscience has sparked interest in understanding the brain’s default mode network (DMN). One particular mental activity that has been identified to recruit such a network is mind-wandering, which points to the functional aspect of mind-wandering as a default system. However, the phenomenological aspect of mind-wandering has been missing in the literature on brain imaging. To tackle this issue, we adopted online thought sampling while participants underwent a simple fixation task over multiple sessions in the scanner. During 10 h of scanning of each participant, over 200 mind wander episodes were labelled in each participant. With linear support vector machine classification on mind-wandering episodes with exclusive sensory content, we found that decoding accuracy in content-corresponding sensory cortices was significantly higher, indicating the neural bases of the phenomenology of mind-wandering. Unique patterns in classification were revealed in different individuals, pointing to individual variances in our phenomenal experiences.

## 1 Introduction

The task free approach in brain imaging has been utilized to a greater and greater extent in the literature owing to its simplicity to implement and its power to extract rich information about brain networks. In particular, during resting-state scans when participants were not performing a task, up to 17 independent networks were parsed and identified (Yeo et al., 2011). In all these networks, the default mode network (DMN), including the medial prefrontal cortex, the temporo-parietal junction, and the posterior cingulate cortex, stands out as a peculiar infrastructure (Fox and Raichle, 2007; Raichle et al., 2001). DMN is most activated when participants are at rest and is most deactivated when participants are on-task and concentrating. Such characteristics of DMN have been linked to the dynamics of our experience when we are not orienting our attention to external stimuli and tasks. In reality, our mind is rarely completely at rest; instead, it wanders. To bridge DMN and mind-wandering, an early approach by Mason et al. (2007) showed that experimental blocks that had higher probability to incur mind-wandering showed stronger activation in the DMN. Furthermore, participants’ propensity for mind-wandering was positively correlated with the signal changes from novel to practiced blocks, suggesting that DMN activation reflected the individuated inclination of deviating from the ongoing task. Using a task-free (i.e., resting state) approach and a post-scan questionnaire, Gorgolewski et al. (2014) were able to look into the self-generated thoughts and their corresponding brain activities during the resting scan. For instance, thoughts about the future were associated with a network encompassing the primary visual cortex.

However, such an approach hardly pinpointed *when* each mind-wandering episode actually occurred but instead relied on the averaging power of the block design, which unavoidably mixed up different mind-wandering episodes and even non-mind-wandering episodes. To cleanly extract each mind-wandering episode, researchers have begun to use *online thought sampling*, which demands that participants to report if they were mind-wandering immediately before the probe. Online thought sampling has allowed researchers to directly extract the mind-wandering episodes and compare them to non-mind-wandering episodes to quantify mind-wandering-specific brain activities. Utilizing such an approach, Christoff et al. (2009) reported that both DMN and the executive network are involved in mind-wandering episodes.

Though the neuroscience community has stepped closer to the functional structure of mind-wandering in the brain, the rich *phenomenology* of mind-wandering is largely missing from the literature. However, more and more studies have started to show that the sensory content and functions of mind-wandering are highly intertwined. For instance, past-oriented mind-wandering has been shown to precede unhappy thoughts in daily life (Killingsworth and Gilbert, 2010). Lab-based experiments have shown the intricate relationship between mind-wandering content and mood from another direction: for instance, when participants were in an unhappy mood, their mind-wandering was more likely to dwell on the past (Smallwood & O'Connor, 2011).

Mind-wandering as a private, subjective mental event has high personal relevance. This phenomenological quality of mind-wandering, however, creates a difficult condition for quantifying the content at the neural level since most human brain imaging techniques rely on similar neural responses toward the same sensory stimulus consistently elicited across participants. A recent fMRI study of experienced mindfulness practitioners reported that around 20% of the reported mind-wandering episodes contained imagery (Ellamil et al., 2016). If these mind-wandering episodes could be caught online and sorted out based on their sensory content, fMRI could be a powerful technique to look into the corresponding brain activities.

In recent years, utilizing a machine learning approach to predict the occurrence of mind-wandering episodes has become popular. For example, combining fMRI and pupil data, Mittner et al. (2014) showed that changes in the functional connectivity in DMN as well as the pupil diameter could be used to accurately classify mind wandering episodes. Unlike approximate one-to-one mapping between a perceptual stimulus and brain response, the unpredictable nature of mind-wandering makes a typical stimulus-response experiment unrealistic. However, a recent study using a small pool of participants (n = 3) has shown successful decoding of sensory content in dreams (Horikawa et al., 2013), which involve involuntary and unpredictable phenomenal experiences. In Horikawa et al.’s (2013) study, each participant underwent hours of scanning so that their dream content could be acquired over multiple probes and multiple scan sessions. Similarly, another fMRI study (Kühn et al., 2014) trained a participant to report her inner speech and then conducted a total of 4 h of scanning across 9 different sessions. The findings showed increased activity in the regions (e.g., inferior frontal gyrus) associated with speech processing during the inner speech episodes. These studies have laid a solid methodological foundation for the current study to investigate phenomenal experiences in mind-wandering.

In the current study, we aimed to bridge our rich phenomenology in mind-wandering and its neural correlates. To ensure that we can capture and quantify sufficient intra-participant mind-wandering sensory content, we adopted a similar approach and recruited a small sample of participants (n = 3) with each participant undergoing 10 h of scanning to acquire >200 mind-wandering episodes. Through online thought sampling, each mind-wandering event was labelled with specific mental content from immediate and comprehensive subjective reports. We expected mind wandering episodes containing sensory content to recruit corresponding sensory cortices. This hypothesis prompted us to narrow down our search in sensory regions that a tight correspondence between an individual’s experience and the region activity has been established, such as the fusiform face area (FFA; Kanwisher et al., 1997), the parahippocampal place area (PPA, Epstein and Kanwisher, 1998), and the lateral occipital complex (LOC, Malach et al., 1995). We expected that mind-wandering episodes containing a specific class of visual stimulus (e.g., faces) will recruit a corresponding region (e.g., FFA), reflected by above-chance decoding accuracies in classifying participants’ visual experience (e.g., faces vs. objects).

## 2 Materials & methods

### 2.1 Participants

Three participants (age range: 25–29) from the Duke-National University of Singapore (Duke-NUS) Medical School community were recruited (1 male) and took part in the six-session study. Each session lasted 2 h. All participants reported being free of any neurological, psychiatric, or sleep disorders. They had normal or corrected-to-normal vision. The experiments were approved by the institutional review boards at the National University of Singapore. All participants gave written informed consent prior to the experiments and were reimbursed with $35/session.

### 2.2 fMRI experiment

#### 2.2.1 Design

Scanning was performed using a 3T Siemens Prisma scanner (Siemens, Erlangen, Germany) at the Duke-NUS Medical School, Singapore. Functional MRI runs were acquired using a gradient echo-planar imaging multiband sequence (TR 1.06 s, TE 32 ms, FA 61°, FOV, 1980 × 1980 mm, 2 × 2 mm in-plane resolution). Thirty-six slices were collected with a 12-channel head coil (2.0 mm thickness). Slices were oriented roughly parallel to the AC-PC with the whole brain being covered. A T1-weighted anatomical image was also acquired and later used for co-registration (TR 2.3 s, TI 900 ms, FA 8°, FOV 256 × 240 mm, 192 slices, 1 × 1 × 1 mm). For the main experiment, each participant took part in approximately 10 runs per session with 5 sessions in total. An additional functional localizer session was collected.

### 2.3 Regions of interest (ROI) localization

To localize the striate cortex (V1), three runs of retinotopic mapping scans were collected. The retinotopic mapping consisted of six 20-s blocks each flanked by 20-s fixation. Stimuli were presented in three experimental conditions. Each condition was repeated twice in a single run. The conditions were presented in a pseudo-randomized order across all three blocks. In the retinotopic mapping scans, flashing checkerboard wedges were presented in each condition. In the horizontal condition, two wedges subtending 10° from the central fixation were presented along the horizontal meridian. Similarly, in the vertical condition, the two wedges were presented along the vertical meridian. In the last experimental condition, four wedges each subtending 30° from fixation were presented along the diagonal axis. During a stimulus block, the color of the central fixation changed between green and red. Subjects were tasked with maintaining fixation at all times and indicating the color of the central fixation cross via button press. The auditory cortex (AC) was localized in another three runs with a similar scan structure of six 20-s blocks with each flanked by 20-s fixation. Three frequency ranges defined the conditions of the auditory localizer: high (2370–5900 Hz), mid (880–2170 Hz), and low (340–870 Hz). In a single stimulus block, pure tones spanning the frequency range were presented in two consecutive 10-s cycles. In a single cycle, the frequency of the tone was linearly increased for 5 s and then decreased for 5 s. Subjects were asked to indicate via button press when the tone’s frequency reached the peak of each frequency range in a block.

Apart from the primary visual cortex and auditory cortex, functional localization of another three regions of interest (ROIs) was based on four independent runs of 20-s blocks (four blocks per category per run) with grayscale images of faces, scenes, common objects and scrambled objects (Kourtzi and Kanwisher, 2001). The fusiform face area (FFA; Kanwisher et al., 1997) was defined as the region of the fusiform gyrus that responded more strongly to images of faces than to images of intact scenes. The parahippocampal place area (PPA) was defined as the region of the parahippocampal gyrus that responded more strongly to images of scenes than to images of intact faces (Epstein and Kanwisher, 1998). Similarly, the lateral occipital complex (LOC) was defined as the region that responded more strongly to images of intact objects than to those of scenes (Malach et al., 1995). All statistical maps were corrected with cluster-thresholding (*p* < 0.05; cluster–forming threshold *p* < 0.01). These lenient thresholds were only used to define the ROIs.

**Fig. 1:**
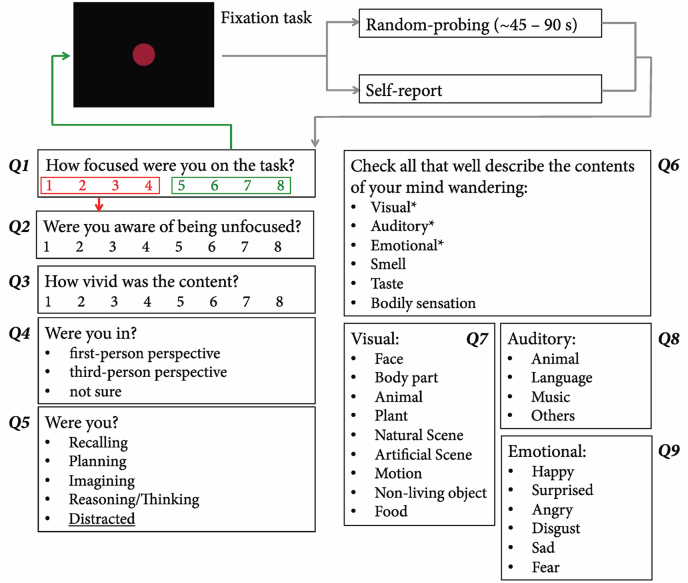
**Experiment procedure.** Each participant underwent 5 main mind-wandering experiment sessions and 1 localizer scan. Each lasted 2 h. In each session, approximately 10 runs were collected. With each run, 9 TRs precedent to the probe was categorized as a non-mind-wandering or a wandering event and labelled with respective sensory content. Five regions including Auditory Cortex (AC, in pink), striate cortex (V1, in purple), FFA (in orange), LOC (in blue), and PPA (in green) were functionally localized in each individual in the separate localizer scan. The multivoxel classification analyses were performed in these sensory ROIs. (For interpretation of the references to color in this figure legend, the reader is referred to the Web version of this article.)

In the main experiment, participants were instructed to do an 8-min fixation task in each run while not thinking about anything in particular. They were told that anything irrelevant to the fixation task would be regarded as mind-wandering. Their eye closure was tracked concurrently to detect drowsiness. If prolonged eye closure was detected twice in a session, the session was abandoned, and a short break was given before the next session began. Two probes were implemented: Random probing occurred every 45–90 s while participants could report mind-wandering anytime if they became aware of it. This time range was determined from our pilot experiments with the same participants. Although we allowed self-reporting in the current paradigm, due to our high sampling rate, none of the participants became aware of the mind-wandering before the probe. The random probing interval was adjusted according to the run-by-run performance with the goal of catching 1 mind-wandering event per minute. If in the previous block, the participant exhibited a mind-wandering rate lower than this threshold, we lengthened the temporal gap between random probes. On the contrary, if the participant exhibited a higher rate than expected, more probes were delivered (gap <1 min). In total, 350 probes occurred over 50 runs in all participants with approximately 70% of them catching mind-wandering events. Objectively, whether a participant mind wandered was determined on an 8-point scale of the first probe question (“How focused were you on the task?”): 1–4 were deemed a mind-wandering event, which led to a series of questions documenting the content of mind-wandering, while 5–8 were labelled as a non-mind-wandering event and the participant returned to the fixation task immediately (Fig. 1). Participants had unlimited time to report the content of their mind-wandering. Prior to the first scan, all participants were familiarized with the procedure. Table 1 details the individual reports. Please note that the current study only focused on the sensory experiences in mind-wandering with the rest of the content collected for other purposes.

**Table 1:** **Detailed individual online mind-wandering reports.** In total, approximately 350 probes occurred in the span of 50 8-min runs, in which over 200 mind-wandering events were collected. Most reports had overlapped sensory content, while the reports with exclusive sensory content are shown in parentheses and used for analyses. Asterisk denotes content with further subcategories.

	MW 01	MW 02	MW 03
Total probes	346	368	349
MW events	233	231	264
Non-MW events	113	137	85
MW percentage	67.3%	62.8%	75.6%
	Sensory categories		
Visual*	183 (24)	139 (21)	196 (40)
Auditory*	115 (11)	83 (17)	108 (14)
Emotional*	87 (9)	42 (5)	44 (2)
Bodily	24 (6)	35 (11)	40 (8)
Taste	3 (0)	10 (1)	17 (1)
Conceptual	80 (6)	120 (46)	57 (33)
Smell	1 (0)	7 (0)	18 (0)
	Visual subcategories		
Face	132 (25)	74 (12)	109 (17)
Body	32 (0)	50 (4)	102 (7)
Animal	5 (1)	0 (0)	14 (5)
Plant	6 (1)	1 (0)	3 (0)
Natural scene	27 (3)	5 (0)	13 (1)
Artificial scene	99 (14)	75 (7)	12 (0)
Motion	14 (4)	25 (2)	21 (0)
Object	34 (13)	52 (14)	78 (26)
Food	5 (2)	11 (1)	29 (7)
	Auditory subcategories		
Animal	1	2	3
Language	94 (74)	66 (54)	100 (94)
Music	39 (19)	19 (14)	8 (2)
Others	3	1	9
	Emotion subcategories		
Happy	40 (25)	18 (14)	15 (15)
Surprised	12 (8)	0 (0)	14 (13)
Angry	13 (8)	5 (3)	6 (5)
Disgusted	7 (3)	3 (2)	5 (5)
Sad	12 (8)	0 (0)	14 (13)
Fear	23 (17)	5 (5)	1 (1)

#### 2.3.1 Data preprocessing

fMRI data analysis was conducted using *freesurfer v6.0* (http://surfer.nmr.mgh.harvard.edu/) and MATLAB R2018a (The MathWorks, Inc., Natick, MA). The processing steps for both the retinotopic mapping and the experimental runs included motion correction and linear trend removal. The processing for the retinotopic mapping and localizer runs used for later univariate (mean) BOLD responses analysis also included spatial smoothing with a 6-mm kernel. For every participant, all the localizer runs were modeled with a general linear model (GLM). A gamma function with delta (δ) = 2.25 and tau (τ) = 1.25 was used to estimate the hemodynamic response for each condition in the retinotopic mapping and localizer scans and the experimental scans. For the experimental runs, the time courses were obtained with a finite impulse response (FIR) model without assuming a particular hemodynamic response function. The FIR model has been shown useful to identify pre-trial signals (Hsieh et al., 2012).

### 2.4 Mind-wandering event labeling and multivariate pattern analysis

**Fig. 2:**
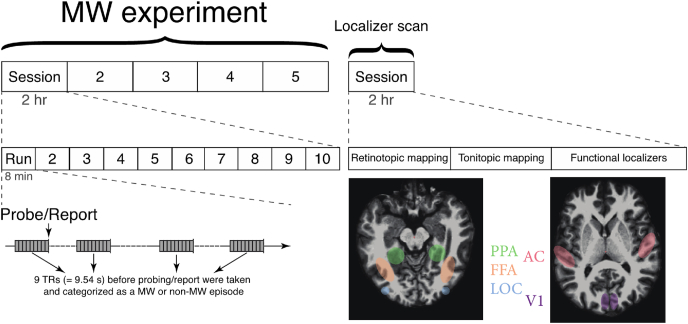
**Online content sampling procedure.** The participant underwent a fixation task in each 8-min run. Every 45–90 s, adjusted according to each individual’s mind-wandering frequency, a question popped out and asked Q1: How focused were you on the task? If the answer was 5–8, this probe was categorized as a non-mind-wandering event, and the participant resumed the fixation task immediately. If the answer was 1–4, the participant proceeded to report a series of questions regarding the mind-wandering content. In Q6, if either of the three answers (visual/auditory/emotional) was chosen, a corresponding subsequent subcategory question would be asked to gather the details of sensory content. Participants were allowed to report their mind-wandering event if they caught it voluntarily, however, no such event took place in our study.

The β value of 9 TRs (9.54 s) preceding the probe or report were extracted and labelled as a mind-wandering or a non-mind-wandering event (Fig. 2, this time window was determined a priori based on Horikawa et al., 2013). Each mind-wandering event was further labelled with corresponding content according to participants’ online reports. We chose two major comparisons: visual vs. auditory and face vs. object. The comparisons made in the final analysis were based on one a priori determination of regions of interest as well as the constraint from the actual data (i.e., available mind-wandering episodes from each participant). Please note that, to ensure only pure sensory categories were included, these events were always exclusive to one category. Furthermore, only categories with >10 episodes were included to avoid biases from a small number of events. For instance, visual events included those events where participants reported having only visual content with no other sensory content (e.g., face only). Thus, although the pre-determined ROIs included PPA, pure scene events were not included in the comparison due to their scarceness in 2 of the participants (number of events both <10). Multivariate pattern analysis was performed in each corresponding ROI of each participant. The β value in each voxel was extracted in each comparing condition in the designated ROI. Furthermore, to ensure that the mean activation difference would not contribute to our multivariate analysis result, β value in each voxel in each condition was normalized against the mean response of the condition before further analysis. A binary linear support vector machine (MATLAB, *fitcsvm*) was built for each comparison. Half of the events from the two comparing conditions were used to train the linear classifier, while the other half were reserved for later testing. Each time, an accuracy rate was obtained by dividing the hit votes with total testing votes. This iteration was repeated 1,000 times for each comparison. Later, the mean decoding accuracy and 99.9% confidence interval were derived across all iterations. There are two reasons why we chose linear SVM. First, due to the limited amount of imaging data, we erred on the safe side and avoided overfitting of the classifier. Second, we understand that recent brain imaging studies have used non-linear classification on the mind-wandering data. Such an approach can be based on their assumption of the data structure or the goal to achieve better classification/prediction (e.g. Kawashima and Kimano, 2017). In our study, we did not intend to make any assumption of our data structure. Furthermore, our goal was to show that sensory cortices contain critical information corresponding to the phenomenological content of mind-wandering. A ceiling classification performance was not part of this goal.

### 2.5 Baseline establishment

One critical limitation of the current study, which will be faced by almost any future mind-wandering studies, is the impulsive and uncontrollable nature of mind-wandering episodes. This factor posed a major deficit on the decoding technique, as one might be decoding the nuanced differences between two sensory content, such as distinct temporal occurrence. This aspect of the nature of mind wandering episodes led to an imbalance in episode distribution, which could potentially drive the decoding performance. We thus needed a baseline classifier performance to catch the decoding performance driven by the imbalance in the episode distribution. To this end, we randomly shuffled the labels of these sensory content and performed the same SVM analysis. For example, when decoding between visual and auditory episodes in the randomly shuffled condition, in each training, half of the visual episodes were randomly selected and labelled as visual while the other half were labelled as auditory. The same procedure was applied to the auditory episodes. The true baseline classification performance was extracted from this random shuffling decoding. Later, comparisons were made between the accuracy of the correctly labelled episodes and that of the randomly shuffled episodes.

## 3 Results

**Fig. 3:**
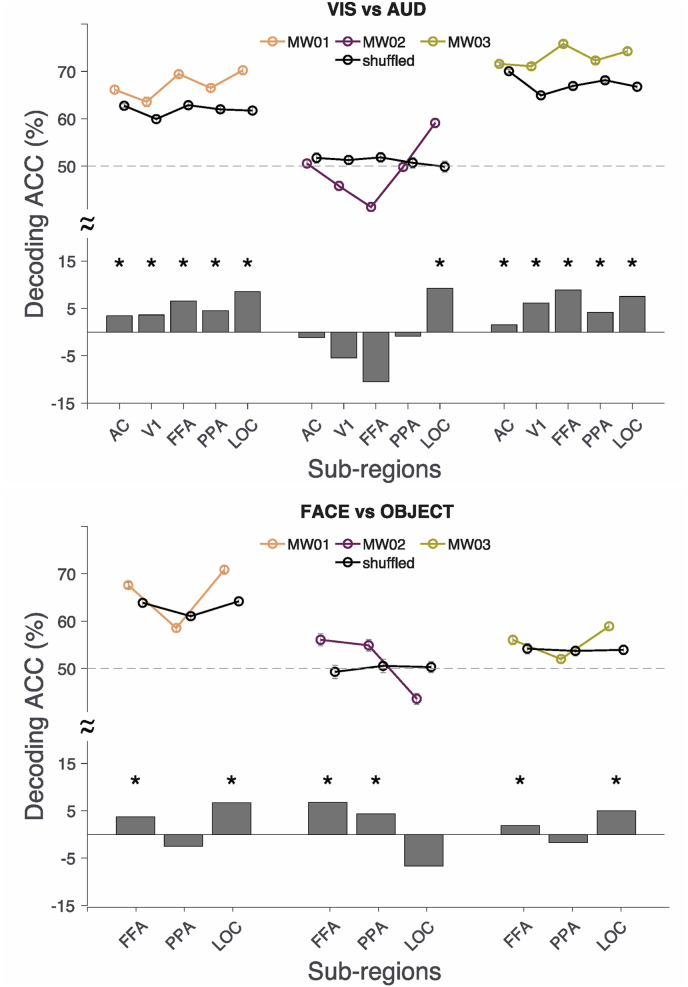
**Decoding accuracy (ACC) of three individuals in sensory ROIs.** The decoding ACC was compared against the baseline where the labels were randomly shuffled. The top half of the figure denotes decoding ACC while the bottom half shows the decoding ACC differences between correctly versus randomly labelled in bar plot. **Top.** Decoding ACC of visual-only vs. auditory-only sensory events in 5 respective sensory regions. **Bottom.** Decoding ACC of face-only vs. object-only sensory events in 3 respective sensory regions. Asterisk denotes significance (all paired t, *p* < 0.00001, significant after Bonferroni correction). Error bars denote 99.9% confidence intervals.

Two major comparisons with enough exclusive sensory content (e.g., visual-only) were made. In mind-wandering events containing visual-only versus auditory-only content, we showed successful decoding in these ROIs (Fig. 3). Importantly, the profile of each individual varied significantly, with some individuals showing above-chance decoding accuracy in all visual and auditory ROIs while others showed better decoding performances only in a high-level visual region (i.e., LOC). In general, better decoding accuracies were found in FFA and LOC, which is consistent with the much higher percentage of mind-wandering episodes containing faces and objects (Table 1). In mind-wandering events containing face-only versus object-only content, we utilized PPA as the baseline region and expected to see chance performance. Again, individuals showed distinct decoding patterns in FFA and LOC, underscoring the vast individual differences of subjective phenomenology in mind-wandering. Across all individuals, successful decoding in FFA was observed with different participants showing distinct decoding accuracies in LOC. The relatively more consistent decoding performance in FFA may suggest a strong bias of mind-wandering content towards human faces (Table 1) and less variance in physical properties across distinct faces, as compared to objects in this pairwise comparison.

Although in the mind-wandering episodes extracted in our study, no vividness difference was observed across individuals (8-point scale, 1 is not vivid at all, and 8 is extremely vivid: MW 01: 1.69; MW 02: 2.29; MW 03: 2.23). We observed individual differences in a post-experiment vividness of visual imagery questionnaire (VVIQ. Marks, 1973). In the VVIQ, participants were asked to answer 16 questions about their visual imagery ability (e.g., Consider a friend’s face that comes before your mind’s eye.) at the scale of 1–5 with the minimum score of 16 and maximum score of 80. These individuals did show differences (eyes closed, MW 01: 72; MW 02: 59; MW 03: 69), suggesting that the ability to generate imagery varied. Another limitation of the current study is the lack of self-caught mind-wandering episodes, which may contain distinct sensory experience profiles. However, there is a rich literature of imagery with explicit meta-awareness (e.g., active visual imagery) showing that specific sensory content recruits sensory regions as if participants were having veridical perception driven by external stimuli (for a review, see Kosslyn et al., 2001). In principle, these results were similar to our findings here. Future work is required to examine how the involvement of meta-awareness in mind-wandering alters the relationship between phenomenology and sensory cortex recruitment.

## 4 General discussion

Our study serves as one of the first brain imaging studies to directly probe the phenomenology of mind-wandering, showing that sensory cortices are indeed activated differently by distinct mind-wandering content. This finding is supported by superior multivoxel classification performance in the corresponding sensory regions when distinct mind-wandering sensory content are decoded. Importantly, each individual exhibits a unique profile of sensory cortices involvement. These individual differences suggest several key features of mind-wandering. For example, the same content (e.g., face) itself might vary significantly in each individual (Marcusson-Clavertz et al., 2016). Furthermore, the post-experiment vividness of visual imagery questionnaire showed distinct propensity to generate visual imagery in the participants. Such differences in visual imagery may also propagate to other modalities. In fact, a previous study has shown concurrent suppression of auditory cortex activation during active visual imagery (Amedi et al., 2005). Importantly, such suppression negatively correlated with VVIQ scores, indicating that people with vivid visual imagery showed stronger concurrent suppression on the irrelevant auditory processing. Our data suggest that a similar modality-separation mechanism could occur in mind-wandering, as participants with higher VVIQ scores showed better decoding performance differentiating visual versus auditory content in the corresponding visual and auditory regions.

Our study highlights the importance of individual-based experimentation and analysis when the core research interests concern personal, private subjective experiences. This approach is distinct from the convention of seeing individual differences as a noisy factor (Kanai and Rees, 2011). A large-scale study is needed to directly assess (1) the interplay between different modalities and sensory content in mind-wandering and (2) the role played by individual propensities to generate mind-wandering. Recently, a single-participant study (Laumann et al., 2015; Poldrack et al., 2015) conducted over a one-year period documented dynamic changes particularly in the functional connectivity of the visual and somato-motor networks in the resting-state scan, which is similar to the mind-wandering condition in the current study. Critically, this finding is largely inconsistent with an inter-subject study that showed the least variability in the sensory-motor and visual regions (Mueller et al., 2013). On the other side of the same coin, an individual-based approach also reveals the hurdle to overcome when researchers intend to study a phenomenon that varies tremendously individually. A priori clustering of participants with similar characteristics could be a foreseeable approach to reduce inter-individual variances and observe the common neural mechanism at the group level.

Another difficulty of studying an individual’s phenomenal experience is the non-extensive nature of individuals’ subjective reports, which leads to a never-ending philosophical debate about the richness of our subjective experience. Supporters of the rich account postulates that our phenomenology overflows our reportability and working memory capacity, and our conscious experience is richer than typical report paradigms can extract (Block, 2011). On the other hand, proponents of the sparse account argue that the limitations of our working memory capacity and the poor quality of our subjective reporting indicate our limited phenomenological content (Cohen and Dennett, 2011). Whether our phenomenology indeed overflows or accurately corresponds with how much information we can select and hold at a given moment, both camps will agree that the subjective report of our conscious experience is heavily gated by various factors, including memory decay, attention selection, etc. In other words, these reports can be - and very often are - imperfect. This very same logic can be applied when we study mental experiences such as mind-wandering: Can we ever obtain comprehensive phenomenal experiences from their reports? A recent “no-report” paradigm has offered a potential solution by first establishing a one-to-one relationship between a percept and a quantifiable objective measure, such as eye movement (Tsuchiya et al., 2015). However, overt behaviors may sometimes have a one-to-multiple relationship to distinct percepts or even subliminal processing (Spering and Carrasco, 2015). A relevant point worth noting is the potential distortion and disruption of mind-wandering episodes in the lab-based experiment, which could change the nature (e.g., frequency/content/vividness/etc.) of the phenomenal experiences per se and therefore may not be completely generalized to daily mind-wandering.

Recently, a heated debate about distinguishing mind-wandering with and without intention has surged (see a review, Seli et al., 2016). As Giambra (1995) pointed out, mind-wandering is not always involuntary but can instead be triggered by voluntary shifts in attention. A recent study showed that intentional mind-wandering contained more future-oriented content and was reported to be more specific than unintentional mind-wandering (Seli et al., 2017). Another recent neuroimaging study also suggested that brain anatomy and connectivity differed for individuals who reported different levels of deliberate/spontaneous mind-wandering (Golchert et al., 2017). How intentionality interacts with the phenomenology of mind-wandering and its neural correlates is beyond the scope of the current study, and future research is needed to disentangle these components.

The current study combines online thought sampling and brain imaging, extending our neuronal understanding of mind-wandering from “functional” to “phenomenological.” Our findings show that distinct sensory content generated by mind-wandering recruit corresponding sensory cortices, highlighting the commonalities of the neural correlates of the phenomenology in mind-wandering, mental imagery (Kosslyn et al., 2001), and stimulus-driven perception (Kanwisher et al., 1997). As the neuroscientific study of mind-wandering has bloomed in the past decades, the research has advanced tremendously and revealed general brain networks underlying this subjective experience (Christoff et al., 2016). Our study further provides a critical missing puzzle of mind-wandering study by showing the neuronal bases of the rich phenomenology when our mind wanders.

## Author contributions

Conceptualization, S.-M.H. and P.-J. H.; Methodology, S.-M.H. and P.-J. H.; Software, S.-M.H.; Investigation, S.-M.H.; Writing – Original Draft, S.-M.H.; Writing – Review & Editing, S.-M.H. and P.-J. H.; Supervision, P.-J. H.

## Declaration of competing interest

The authors declare that they have no known competing financial interests or personal relationships that could have appeared to influence the work reported in this paper.
